# Multifactorial dysphagia: Azygos vein aneurysm (AVA) and esophagogastric junction outflow obstruction (EGJOO)

**DOI:** 10.1016/j.ijscr.2021.106017

**Published:** 2021-05-26

**Authors:** Scott Morton, Andrew D. Grubic, Shahin Ayazi, Satish C. Muluk, Hiran C. Fernando, Blair A. Jobe

**Affiliations:** a Esophageal Institute, Department of Surgery, Allegheny Health Network, Pittsburgh, PA, United States of America; b Division of Vascular Surgery, Department of Thoracic and Cardiovascular Surgery, Allegheny Health Network, Pittsburgh, PA, United States of America; c Division of Thoracic Surgery, Department of Thoracic and Cardiovascular Surgery, Allegheny Health Network, Pittsburgh, PA, United States of America

**Keywords:** Dysphagia, Azygos vein aneurysm, Embolization, Esophagogastric junction outflow obstruction (EGJOO), Myotomy

## Abstract

**Introduction:**

Vascular impingement of the esophagus is a rare cause of dysphagia, and is most commonly due to aortic arch anomalies such as arterial lusoria. Dysphagia resultant from venous compression is even further less likely.

**Presentation of case:**

We present a highly unusual case of dysphagia secondary to a large aneurysm of the azygous vein near its confluence with the superior vena cava, which was managed with endovascular modalities. Despite initial treatment success, patient reported some intermittent solid food dysphagia, and was also found to have esophagogastric junction outflow obstruction (EGJOO) on high resolution impedance manometry (HRIM) which was successfully managed with surgical myotomy and partial fundoplication.

**Discussion:**

The azygos vein has an intimate anatomic relationship with the esophagus as it traverses the posterior mediastinum. Because of this anatomic association, the azygos vein may present a point of esophageal obstruction in the setting of significant pathology.

**Conclusion:**

This case highlights the possibility of multifactorial causes of dysphagia, and that HRIM is a key aspect of this workup. Additionally we discuss the pertinent anatomy, diagnosis, and treatments for azygos vein aneurysm and EGJOO.

## Highlights

• Vascular impingement of the esophagus is a rare cause of dysphagia.
• This type of dysphagia is most commonly due to aortic arch anomalies such as arterial lusoria.
• The azygos vein may present a point of esophageal obstruction in the setting of significant pathology.
• Endovascular therapy in selected patients can provide resolution of dysphagia in patients with azygos vein aneurysm.
• There is possibility of multifactorial causes of dysphagia, and high resolution manometry is a key aspect of this workup.

## 1 Background

External vascular compression is a rare, but critical differential diagnosis for dysphagia, and is usually associated with arterial anomalies. Of these, arterial lusoria is most common, and occurs when an aberrant right subclavian artery originates from the aortic arch distal to the left subclavian artery, coursing posterior to the esophagus. Arterial lusoria may be associated with other vascular anomalies including truncus bicaroticus, Kommerell's diverticulum, aneurysm, and right-sided aortic arch. In the 7–10% of patients who manifest symptoms from these anomalies, the condition is referred to as dysphagia lusoria [1].

On the contrary, venous abnormalities are even more unlikely to produce mass-effect, due to their relative amuscular structure and compressibility. The Azygos Vein (AV) originates as the confluence of the upper lumbar and right subcostal veins prior to entering the thorax. It courses closely with the esophagus and thoracic duct through the posterior mediastinum and receives left-sided venous contributions from the hemiazygos and accessory hemiazygos veins. The AV arches over the esophagus anteriorly prior to its termination in the superior vena cava (SVC) [2]. Given its intimate anatomic relationship with the esophagus, the AV has the potential to produce dysphagia symptoms in the setting of significant pathology.

Azygos vein aneurysm (AVA) was identified on autopsy by William Osler in 1915 and was later described by William Walker in 1963 [3]. Radiographically, the normal sized azygos vein (AV) is typically ≤1 cm however caliber can vary between anatomic regions and volume status. Although there is no formal, diagnostic size criteria for AVA, focal dilation of ≥2.5 cm at the AV body and ≥ 3.75 cm at the AV arch have been purposed [4].

AVA can result from elevated venous pressures in the setting of congestive heart failure, cirrhosis, pregnancy, as well as central venous occlusion with thrombus or tumor. Additionally, structural alterations of the vessel wall may potentiate aneurysmal (or pseudoaneurysmal) dilation, and have been associated with connective tissue disorders, trauma, or infection. Despite all of these potential etiologies, a 2017 review of 57 published cases found that AVA was idiopathic in the majority of instances. The same review also noted that presentation was primarily incidental on imaging, with only a minority presenting with chest discomfort, dyspnea, or cough. Interestingly only one patient was noted to have mild dysphagia as an ancillary symptom. Here we present the case of a patient who presented with progressive, dysphagia and odynophagia, which was ultimately discovered to be secondary to AVA compression of the esophagus. To our knowledge this is the first reported case of this dramatic clinical manifestation of AVA in the current literature. This work has been reported in line with the SCARE 2020 criteria [5].

## 2 Case presentation

Patient is a 73-year-old female with a history of chronic obstructive pulmonary disease, gastroesophageal reflux disease, diverticulitis, hyperlipidemia, and osteochondroma of the right scapula which was resected as an early teenager. She developed progressive dysphagia and odynophagia, as well as chest tightness in late 2019, prompting evaluation. Multiphase CT scan showed a 5.5 × 5.0 cm aneurysm of the azygous vein at the arch extending near its termination in the superior vena cava, resulting in displacement of the carina anteriorly as well as the esophagus toward the aorta and spine. Barium swallow revealed significant external compression of the esophagus in the region of the aneurysm with adjacent dysmotility and delayed passage of a 13.5 mm barium tablet.

**Fig. 1:**
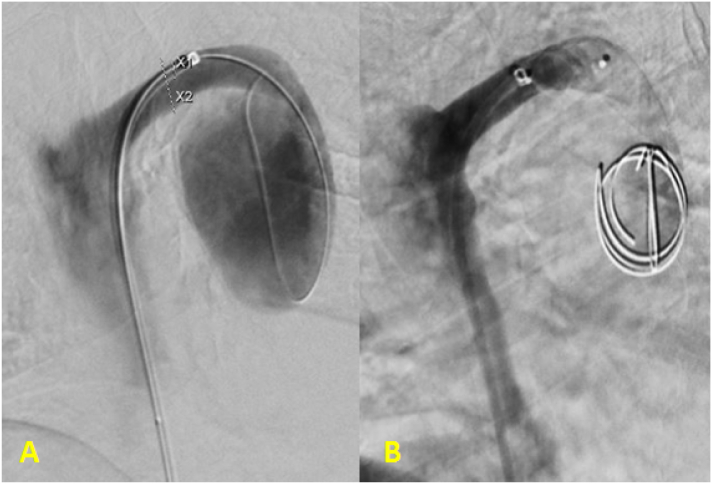
Venography showing the perfused azygos vein and aneurism (A) and the aneurysm and vein following coil embolization and vascular plug placement (B).

**Fig. 2:**
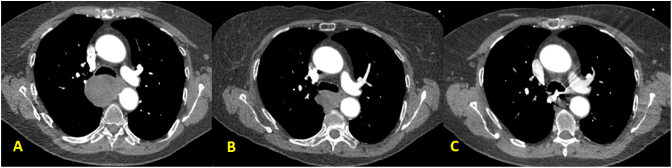
CT Angiogram showing regression of azygos vein aneurysm size at one-month 4.6 × 4.2 cm (A), three-months 3.3 × 2.3 cm (B), and six-months 1.0 × 2.4 cm (C) following endovascular treatment.

Patient was referred to vascular surgery and underwent 20 × 30 mm helical hydrocoil embolization of the aneurysm as well as 12 mm Amplatzer plug occlusion of the AV inflow via a right femoral endovascular approach (Fig. 1). Procedure was uncomplicated and patient recovered quickly. In the following weeks, she reported a drastic improvement of symptoms particularly with dysphagia. At one-month follow up, there was complete thrombosis of the AVA with size decreased to 4.6 × 4.2 cm on CT angiogram (Fig. 2A) as well as significantly improved contrast passage on barium swallow.

**Fig. 3:**
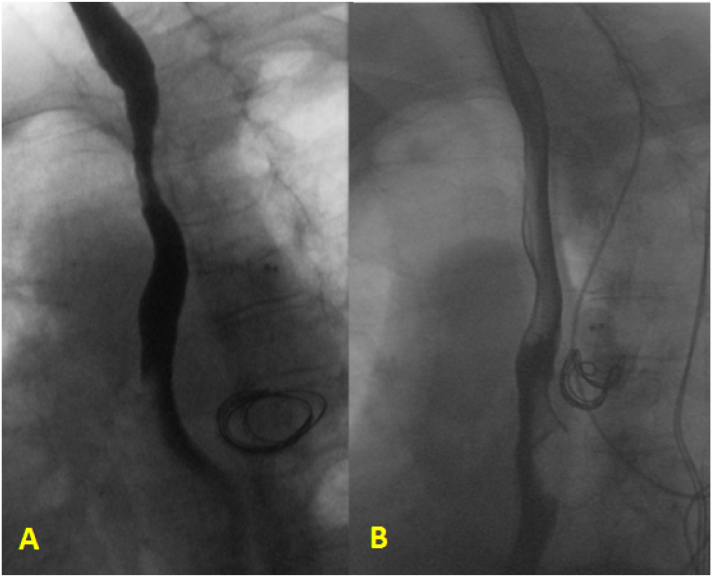
Barium swallow showing improved luminal patency and motility at one-month (A) and six-months (B) after endovascular treatment.

Three months after the procedure, CT angiogram indicated that the AVA further reduced to 3.3 × 2.3 cm (Fig. 2B). On upper endoscopy, only mild external compression was observed at 28–30 cm from the incisors, and did not result in significant luminal narrowing. At six-month follow up, CT angiogram found that the AVA size continued to decrease to 1.0 × 2.4 cm. Barium swallow at that time revealed minimal esophageal compression with resolution of the previous delayed transit and adjacent dysmotility (Fig. 3).

**Fig. 4:**
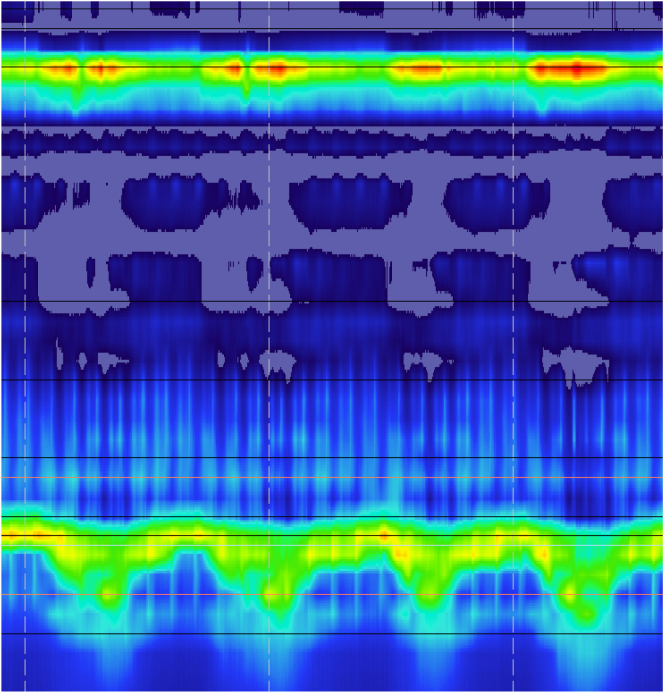
High resolution manometry tracing performed following endovascular intervention showing no evidences of extra-luminal compression at the level of azygus vein aneurysm.

**Fig. 5:**
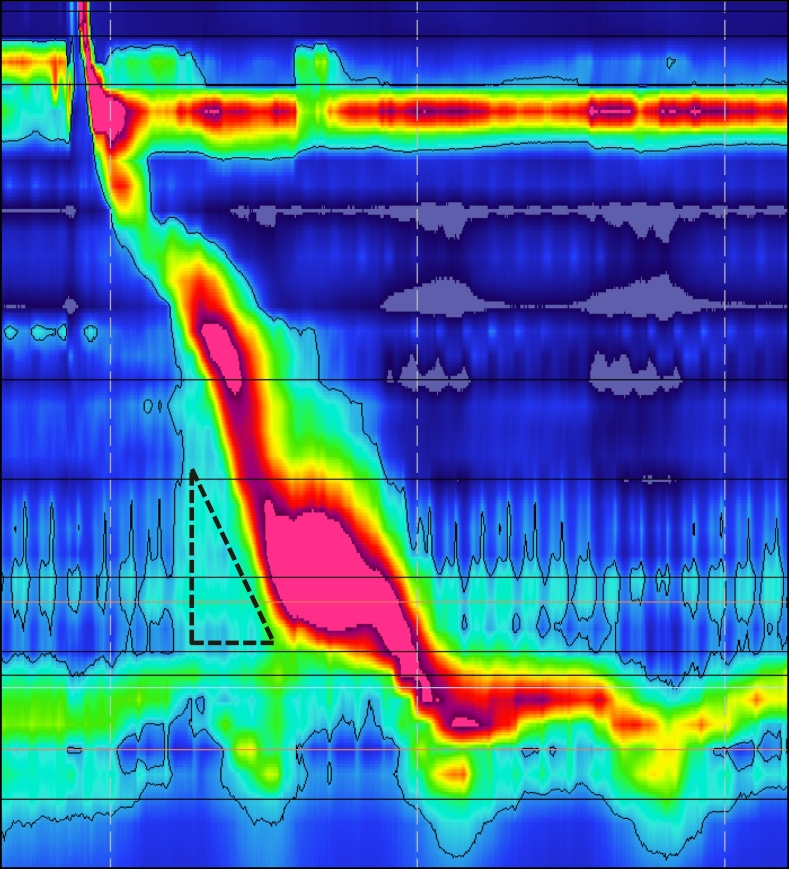
A sample contraction from high resolution impedance manometry after endovascular intervention showing integrated relaxation pressure (IRP) of 19 mmHg and raised intrabolus pressure (iBP) marked by a dotted triangle. Isobaric contour is set at 15 mmHg in this manometry tracing.

Despite her initial improvements, patient reported intermittent solid food dysphagia. She went on to complete further workup of dysphasgia and gastroesophageal reflux disease. . She was found to have a 2 cm hiatal hernia, however 48-h Bravo returned normal. High resolution impedance manometry (HRIM) did not show any manometric evidences of extra-luminal compression in the esophageal body at the level of AVA (Fig. 4). Of note, HRIM revealed elevated median integrated relaxation pressure (IRP) of 19 mmHg with increased intrabolus pressure (iBP), suggestive of esophagogastric junction outflow obstruction (EGJOO) (Fig. 5). She subsequently underwent robotic assisted Heller myotomy with anterior 180° Dor fundoplication. Procedure was uncomplicated, and the patient recovered well. Currently patient remains completely free of dysphagia and is tolerating a regular diet. The Eckardt score of the patient was 12 on her initial presentation and markedly improved to 0 following these interventions.

## 3 Discussion

In the presented case, the patient had a considerably large aneurysm of the azygos vein arch. This anterior location is critical as the esophagus was forced against the T4 vertebral body, likely explaining her dysphagia-predominant symptoms. The region is also notable due to the fact that the AVA occurred in the segment of vein between a typical valve of the AV arch [6] and the insertion into the superior vena cava. Theoretically elevated pressures in the right atrium against these AV arch valves could potentiate aneurysmal dilation in this region. Interestingly patient did not have known systemic hypervolemia, pulmonary hypertension, or right-sided heart failure, highlighting its idiopathic nature.

Treatment of azygos vein aneurysm encompasses prevention of thromboembolism and rupture as well as alleviation of symptoms. Management has yet to be completely defined, and is variable depending on symptomatology, anatomy, institution, and patient factors. For asymptomatic AVA, a conservative or surveillance approach is utilized in the majority of cases, and has been supported by the American Venous Forum. Due to some rare reports of spontaneous thrombosis and pulmonary embolism, some authors have proposed systemic anticoagulation. While this practice is in direct conflict with any potential for AVA rupture, the risk of spontaneous rupture is primarily theoretical and remains controversial [4].

For symptomatic patients, intervention is generally agreed upon but there is no consensus for threshold or modality. Surgical resection with or without venous bypass has been performed with excellent results. Complete vascular control is required to prevent massive hemorrhage and embolectomy may also be required. Minimally invasive approaches provide less post-operative pain but may prove quite challenging for aneurysms especially near the confluence with the SVC. Additionally, stapling techniques of the thin-walled, aneurysmal vein should be precise to avoid post-operative hemorrhage. For this same reason vigilant fluid and hemodynamic management is also needed. Proponents of resection favor the more definitive nature of the procedure, particularly with smaller and more accessible AVAs [4].

More recently, endovascular approaches have also been shown to also be safe and effective. Several modalities have been employed including stenting, venous occlusion, and embolization. Of these methods, stenting is favored by many due to the ability to exclude the aneurysm while preserving venous patency, but may be limited by anatomic location. Some critics have argued that coil embolization alone does not prevent the risk of future thromboembolism. Additionally there has been some concern that addition of vascular coils to the aneurysm may worsen compressive effect [4].

The decision to proceed with endovascular management in this patient was primarily based on anatomic factors. Given that the large, saccular aneurysm approached the SVC, minimally invasive vascular control would have been difficult. Distal ligation may have encroached on the SVC potentially serving as a nidus for future thrombosis or stricture. Additionally, patient's history of chronic obstructive pulmonary disease certainly contributed to surgical risks. From an endovascular standpoint, conservative coil embolization was performed in the AVA sac until thrombosis was initiated, and the AV inflow was occluded with a vascular plug. The intention of this combination approach was to minimize foreign material within the aneurysm, halt flow, and reduce risk of embolization, which could provide the best scenario for future regression. Ultimately cessation of AV flow and induction of AVA thrombosis resulted in swift improvement of dysphagia symptoms in addition to marked radiographic evidence of AVA recession.

This case also highlights the possibility of multifactorial causes of dysphagia, and that HRIM is a key aspect of this workup. Esophagogastric junction outlet obstruction (EGJOO) is a fairly new manometric designation and includes previously termed hypertensive lower esophageal sphincter (LES) cases. The diagnosis of EGJOO is based entirely on HRIM. Alternatively the diagnosis of hypertensive LES was based on conventional manometry and the diagnosis has therefore primarily shifted to EGJOO and was eventually included in Chicago Classification 3.0 [7,8]. Studies show an approximate incidence of 3–11% on HRIM [9]. There are some investigators who believe that EGJOO is an incomplete expression of achalasia or an early achalasia although the data have been variable at proving this thus far and further investigation with long term follow up is necessary [8].

Diagnosis of EGJOO is based on HRIM with elevated integrated relaxation pressure (IRP) above 15 mmHg and normal peristalsis of the esophageal body, excluding the diagnosis of achalasia [10]. There will also be increased intrabolus pressure proximal to the LES with normal bolus clearance [7,10]. Also of note, during HRIM the IRP will not be useful in the setting of a large hiatal hernia as the measurements of integrated relaxation pressure will be artificially higher using the intra-hiatal hernia pressure rather than true intraabdominal and intragastric pressure. A patient with elevated IRP and without the findings of large hiatal hernia or lack of peristalsis can therefore be identified as having EGJOO [10].

It is important to mention that the presented patient was diagnosed with EGJOO based on the Chicago Classification 3.0 definition. Recently the diagnosis has changed in Chicago Classification 4.0 was refined to better define true EGJOO. The new definition requires that IRP remain elevated in both the upright and supine positions, and at least 20% of swallows demonstrate elevated intrabolus pressure. Additionally mechanical obstruction must be excluded by timed barium swallow and/or endoscopy [11].

Myotomy remains the most definitive treatment for true EGJOO and can be accomplished by surgical or endoscopic approaches. Prior to the advent of the per-oral endoscopic myotomy (POEM) procedure, Patti et al. reviewed 208 patients with disease of the esophagus to identify whether laparoscopic Heller myotomy with Dor fundoplication (LHM-DF) was a success at treating the disease. While the treatment was very successful, and even quoted to be the procedure of choice, their study only identified two patients total with EGJOO [12]. A case report by Pereira et al. showed a complete response of GEJOO to LHM-DF, suggesting that this would be an ideal operation for these patients [13]. A subsequent review by Hungness et al. enrolled 112 patients, 9 with EGJOO, for the POEM procedure and close follow up to assess resolution of their disease. One patient had recurrence of symptoms at 2 years with no additional treatment and the other at 3 months requiring dilation and subsequently LHM-DF. While the numbers overall are low, this would indicate that both LHM-DF and POEM are successful procedures to perform on EGJOO patients [14].

Although POEM was considered for this patient, decision for surgical myotomy was significantly impacted by the prior AV embolization. Using the rationale that the occluded AV may increase collateral venous flow in the distal esophagus, it was felt that a surgical approach would reduce potential bleeding complications. Additionally, hiatal hernia repair and restoration of the antireflux barrier would provide more durable control of reflux.

## 4 Conclusions

Aneurysms of the azygos vein are extraordinarily rare and presentation is often incidental on imaging. Of symptomatic patients, dysphagia is typically mild or not present. A large AVA near the confluence with the SVC may produce significant dysphagia due to esophageal compression against the spine. Endovascular therapy in selected patients can provide resolution of dysphagia and significant radiographic regression in patients with azygos vein aneurysm. Additionally it is important to consider alternative and multiple etiologies for dysphagia, particularly in patients whom have incomplete response to initial treatments.

## CRediT authorship contribution statement

Equal contribution by all authors.

## Declaration of competing interest

The authors have no relevant conflict of interest to disclose.
